# Assessment of the fecundity of deep-sea corals in southeastern Brazil

**DOI:** 10.7717/peerj.19525

**Published:** 2025-06-03

**Authors:** Nathália Bastos, Carolina Siqueira Safra Terra, Márcio Alves Siqueira, Lourença Helena de Oliveira Vieira, Caio de Lima Mota, Halesio Milton Correa de Barros Neto, Guarani de Hollanda Cavalcanti, Ricardo Coutinho

**Affiliations:** 1 Departamento de Biotecnologia Marinha, Instituto de Estudos do Mar Almirante Paulo Moreira-IEAPM, Arraial do Cabo, Rio de Janeiro, Brasil; 2 Centro de Pesquisa e Desenvolvimento da Petrobras-CENPES, Rio de Janeiro, Rio de Janeiro, Brasil

**Keywords:** Reproduction, Scleractinia, Cold-water coral ecosystem, Southwestern Atlantic, Oil and gas

## Abstract

Deep-sea corals have been facing several anthropogenic threats worldwide, making it increasingly important that studies better understand their reproductive biology and associated cycles. This study described the fecundity of the main habitat-building scleractinian species in three sedimentary basins of southeastern Brazil in two pre-determined periods over 2 years. These basins are responsible for the most significant oil and gas production on the Brazilian coast. The relation between the number of gametes and the size of the individuals’ polyps determines their fecundity. *Madrepora oculata*, *Solenosmilia variabilis*, *Desmophyllum pertusum* (formerly *Lophelia pertusa*), and *Enallopsammia rostrata* samples were obtained in 2016 and 2017 and histologically prepared to estimate the reproductive effort of these species. Each oocyte development stage was classified and counted to evaluate the reproduction strategies of each species. A single *D. pertusum* polyp (collected in May 2017) showed over 241 thousand oocytes and a 167.9 oocytes/mm^3^ fecundity, typical of periodic reproduction. *S. variabilis* had the highest average fecundity (53.6 ± 10.7 oocytes/mm^3^) of the species with a continuous gametogenic cycle. *M. oculata* showed 23.5 ± 7.03 oocytes/mm^3^, whereas *E. rostrata* had the lowest fecundity (3.1 ± 0.7 oocytes/mm^3^). Fecundity is inversely proportional to oocyte size, and *E. rostrata* showed the largest oocytes (900 μm), a result aligned with previous studies. Considering the entire sample, the Espírito Santo Basin was the most productive due to its highest average fecundity (followed by the Santos and the Campos Basins) and greatest number of female samples. Notably, 2017 showed the highest average fecundity. The reproductive strategies of organisms play an important role in the ability of species to respond to selective pressures since gamete production (especially oocytes) is energetically expensive and strongly sensitive to environmental conditions. Thus, this study contributes to refining the available data about the life history and resilience strategies of deep-water corals, providing scientific information to manage and conserve these deep-sea ecosystems.

## Introduction

Deep-sea scleractinian corals build highly diverse ecosystems (Lumsden et al., 2007; Brooke & Järnegren, 2013; Soetaert et al., 2016), providing protective and nourishing habitats. Furthermore, they represent a breeding refuge for many commercial fish species (Rogers, 1999; Mortensen, 2000) and shelter a high diversity and abundance of fauna (Beazley et al., 2021). Improving knowledge about coral reproduction is decisive for their conservation, management, and restoration. Therefore, this study addresses the reproduction of these corals by the fecundity of colonies collected inside and outside the reproductive period (Pires, Silva & Bastos, 2014). Reproduction constitutes a parameter with a lower stress tolerance than other vital functions. Therefore, any changes in reproductive activity may indicate environmental changes (Harrison & Wallace, 1990). Reproduction configures the most vital ecological process to the life history strategy of individuals and their populations. It can maintain current populations, provide means for expansion into new areas (Waller et al., 2023), and recover populations damaged by disturbances (which can only be restored by reproductive success) (Fiorillo et al., 2013; Kersting et al., 2013).

The Brazilian continental margin has a great diversity and abundance of azooxanthellate corals, which, according to Pires (2007), has a greater richness than zooxanthellate species. However, due to the challenging logistics to sample these organisms in Brazil, knowledge of the biology and reproduction of deep-sea corals is still scarce (Pires, Silva & Bastos, 2014). According to Kitahara et al. (2020), the fauna of Brazilian sedimentary basins is one of the least known in the world. Over the past three decades, studies on these ecosystems have increased, the findings of which show that various exploitation practices, pollution, climate warming, and ocean acidification threaten coral habitats (Roberts et al., 2009). The species *Desmophyllum pertusum* (Linnaeus, 1758) (a new combination of *Lophelia pertusa* by Addamo et al. (2016)) and *Solenosmilia variabilis* (Duncan, 1873) are widely present along the continental margin in southeastern Brazil. They constitute one of the most important habitat-forming deep water species (Pires, 2007). This research also considers *Madrepora oculata* (Linnaeus, 1758) and the cosmopolitan *Enallopsammia rostrata* (Pourtalès, 1878) as important deep-sea coral mounds off the Brazilian continental margin (Pires, Silva & Bastos, 2014). Recent research has considered *S. variabilis* as the most prevalent colonial species in coral mounds, whereas *D. pertusum* occurs more abundantly in the central waters of the South Atlantic, both regions of the Santos Basin (Carvalho et al., 2023).

Most shallow-water scleractinians are hermaphrodites, unlike most deep-water corals (which are gonochoric) (Harrison, 2011). In a study of reproductive ecology in the North Atlantic, Waller & Tyler (2005) found that *Desmophyllum pertusum* reproduces seasonally and that *Madrepora oculata* shows multiple cohorts of gamete production. *D. pertusum* has a seasonal reproductive cycle and spends much time maturing its gametes (Waller & Tyler, 2005), accumulating many that are ready to spawn at the end of gametogenesis. Pires, Silva & Bastos (2014) studied the biological and anatomical aspects of the gametogenesis of four scleractinian species in the Campos basin, describing the reproduction of *M. oculata*, *E. rostrata*, and *S. variabilis* as continuous throughout the studied year. They found that the reproductive peak of *S. variabilis* occurs from April to September, whereas *D. pertusum* showed a seasonal reproductive peak from May to July. These findings have motivated our assessment of the fecundity of these corals in three sedimentary basins on the Brazilian southeast continental margin.

Fecundity, expressed by the number and size of eggs and/or the occurrence of larvae per polyp, helps to estimate reproductive effort (Harrison & Wallace, 1990) and can consistently indicate the number of yearly reproductive cycles (Babcock, Willis & Simpson, 1994). Environmental conditions can influence sexual processes, and, according to Olive & Garwood (1983), the factors that trigger specific changes in reproductive activities—which can regulate sexual cycles in marine invertebrates—are exogenous (external environmental), interacting with endogenous biorhythms (internal environmental). Thus, knowledge of reproductive processes such as fecundity can indicate stress in corals (Harrison & Wallace, 1990) and help to assess their environmental resilience. Knowing the fecundity of a species and the extent of its variation in space and time is an essential element for biological and ecological studies, thus constituting a basis to establish conservation and management practices (Waller, 2005). According to Kojis & Quinn (1984), species that reproduce throughout the year, such as *Acropora (Isopora) palifera*, would be particularly useful in monitoring programs as they facilitate the assessment of fecundity (as do all other continuously reproducing species, such as *M. oculata* and *E. rostrata*). Reproduction greatly suffers the effects of stress and may cease if animals undergo external pressure (Reynaud & Ferrier-Pagès, 2019). We can link the current structure of populations to their reproductive status investigating fecundity (Bramanti et al., 2019). The main difficulty with this procedure refers to the need to sample a minimum number of mature colonies before they release their gametes. Knowledge about reproductive activity, gamete quality, and fecundity is fundamental to assess the resilience of that ecosystem. The reproductive status of coral populations can serve as a biomarker of the health of individuals (Orejas & Jiménez, 2019).

This study assesses the reproductive effort of the four main habitat-forming corals on the Brazilian coast by evaluating their fecundity inside and outside their known reproductive peak periods in three important sedimentary basins. We determined the fecundity of these corals by counting their female gametes, measuring polyp volume, and relating both measurements. This research compared the number of female gametes and reproductive cycles with the findings of previous studies. We also evaluated sex ratio, size, stages of gamete development, and occurrence of mature spermatic cysts to assess reproductive periods and timing.

## Materials and Methods

### Study area

**Figure 1:**
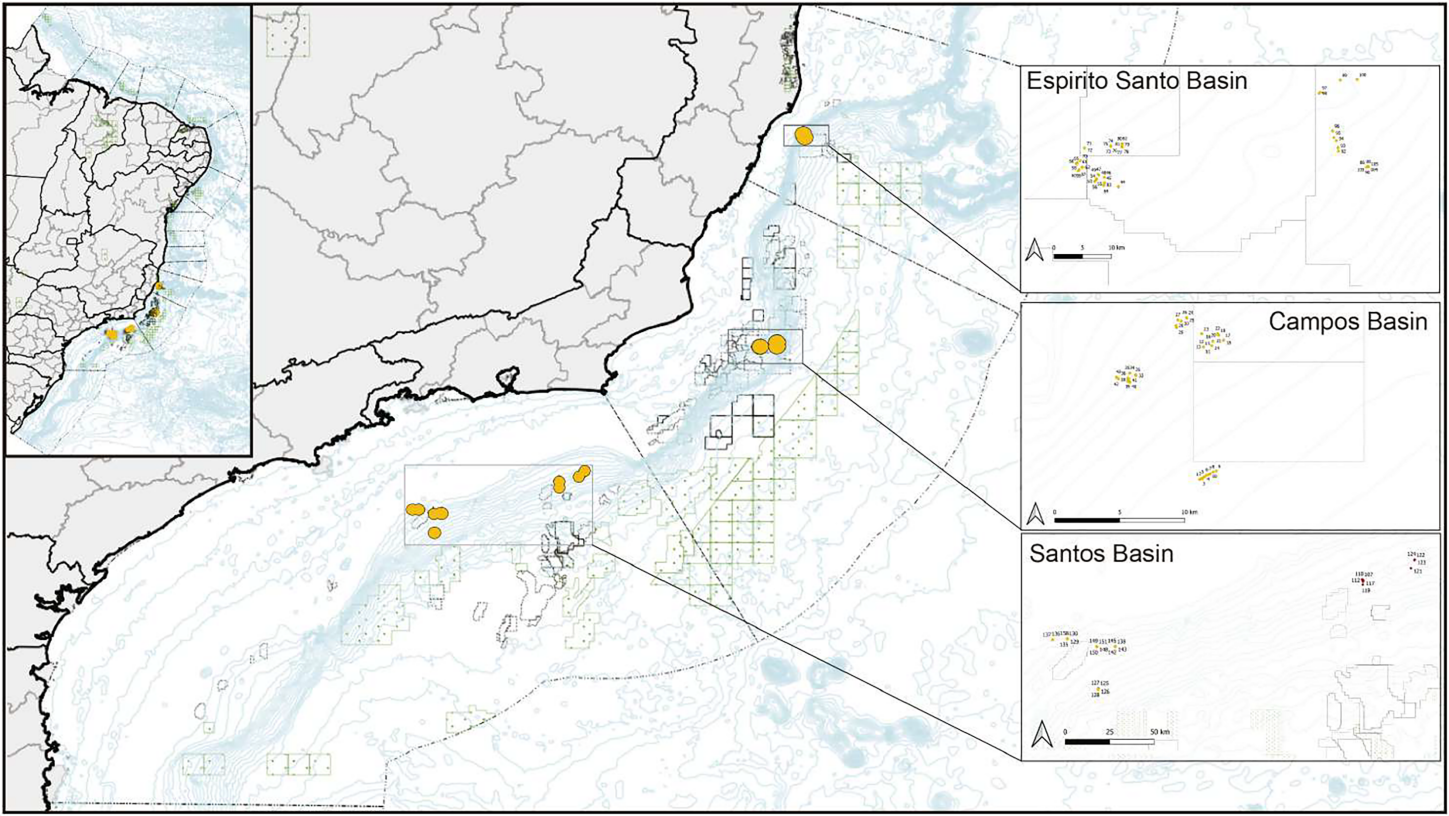
Map of collection sites on the southeastern Brazilian continental margin. Corals were collected in the north in the Espírito Santo Basin, in the center in the Campos Basin, and in the south in the Santos Basin.

Samples were provided by two oceanographic cruises carried out by the Petrobras Research and Development Center under the Sensitive Marine Environments project in the three main sedimentary basins of the Southwest Atlantic (Fig. 1). Our first field campaign occurred from August 31 to October 5, 2016, and the second, from May 27 to August 1, 2017. Coral colony fragments were collected by a remotely operated vehicle. The Espírito Santo Basin (ESB)—the northernmost collection site of the other areas—spans 125,000 km^2^, of which 18,000 km^2^ constitute its terrestrial basin (Fiduk et al., 2003). To the east of the ESB, the physiography shows a widened continental shelf that averages 40 km in its southern portion and reaches up to 240 km in its central-northern portion (França et al., 2007). The Campos Basin (BC) lies on the southeastern margin of Brazil (from 20.5 to 24°S) and spans more than 100,000 km^2^. It lies almost entirely under over 200 m of water. The width of its continental shelf averages 100 km, and its break has an average depth of 110 m from its northern to its southern areas. Its slope spans over 40 km (Viana et al., 1998). The Campos Basin constitutes a large and important oil and gas production area. Its high biological primary productivity site in its water column stems from the resurgence events of nutrient-rich and cold South Atlantic Central Water (Oliveira, Cordeiro & Carreira, 2013). The Santos Basin is located between the parallels 23° and 28° S, occupying approximately 350,000 km^2^ up to the bathymetric elevation of 3,000 m. It is bordered to the north by the Campos Basin and to the south by the Pelotas Basin. Recent research has mapped the bottom topography of the Santos Basin, finding topographic features in its bathyal region (~200–3,000 m depth) that interfere with the flow of the bottom current, forming hard substrates at multiple depths and creating suitable conditions for coral colonization. The most frequent taxa that have been observed in videos recorded by remotely operated vehicles include scleractinians and octocorals (Carvalho et al., 2023).

### Collections

In 2016, colonies were collected in the ESB from August 31 to September 3; in the CB, from September 7 to 10; and in the SB from September 30 to October 5. In 2017, colonies were collected in the ESB from May 27 to June 1; in the CB, from July 20 to 27; and in the SB, from July 29 to August 1 (Supplemental Material). Two hundred and seventeen colonies were analyzed—105 from the 2016 campaign and 112 from 2017. We selected 40 *M. oculata* (*n* = 120 polyps), 77 *D. pertusum* (*n* = 228 polyps), 42 *E. rostrata* (*n* = 126 polyps), and 58 *S. variabilis* colonies (*n* = 174 polyps). Coral colonies were collected from several depth ranges. Collections were performed from 531 to 1,128 m at the CB, from 205 to 892 m at the SB, and from 456 to 949 m at the ESB. Samples were fixed in 4% formaldehyde immediately after collection. Authorization for the collection and transportation of biological material was granted by IBAMA (No. 731/2016).

### Histology

**Figure 2:**
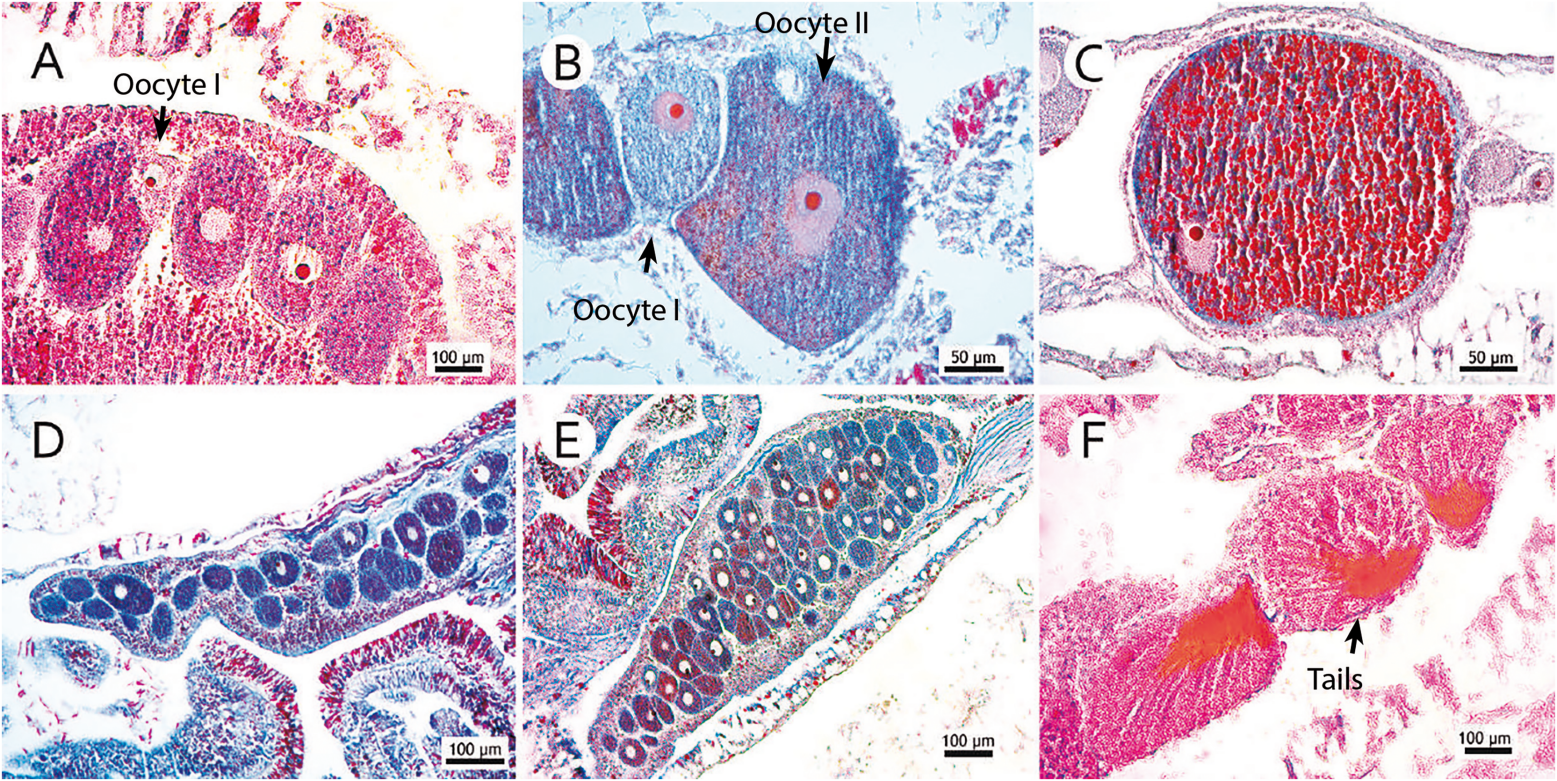
Photomicrographs of three stages of oocyte development, reproductive mesentery, and mature spermatic cysts. (A) *M. oculata* oocyte I, light pink, between developing oocytes to stage II bigger and darker; (B) *S. variabilis* oocyte II, dark blue, and an oocyte I, smaller and lighter, both with a more centralized nucleus; (C) *M. oculata* oocyte III filled with red-stained lipid vesicles and more peripheral nucleus; (D) *D. pertusum* reproductive mesentery with oocytes; (E) *S. variabilis* oocyte sac in a reproductive mesentery; (F) *M. oculata* mature spermatic cysts (stage III) with tails-stained orange.

For histological evaluation, we chose the more robust branches of the colony fragment (indicating the more basal branches in the sample), since coral fecundity depends on age (Harrison & Wallace, 1990; Hall & Hughes, 1996) and thinner branches may indicate peripheral and new structures with polyps out of reproductive age. This study did not evaluate colony morphology and size. The size of the selected fragments spanned up to 10 centimeters and contained a variable number of polyps. Samples were decalcified in a 10% formic acid solution and 5% formaldehyde until their skeletons completely disappeared. Then, three polyps were randomly selected for histological evaluation. The largest (height) and smallest diameters (oral disc) were measured to estimate polyp volume, which was correlated with the number of found oocytes to determine fecundity. Polyps were embedded in paraffin and cut into 7 µm sections. As per Pires, Castro & Ratto (1999), slides were stained with Mallory’s trichrome. All oocytes with apparent nucleoli were counted and measured without repetitions. Photomicrographs of the representative sections were generated by a digital camera (Axio Cam ER c5s Zeiss; Zeiss, Oberkochen, Germany) coupled to an optical microscope (Zeiss Axio; Zeiss, Oberkochen, Germany). Gamete development was classified following Pires, Silva & Bastos (2014), in which stage I represented the beginning of development (previtellogenesis); stage II, an initial vitellogenesis; and stage III, mature oocytes or late vitellogenesis. These matured oocytes are easily distinguished from others due to their high concentration of lipid vesicles forming a lipid-rich yolk. The largest oocyte diameter was measured by a Zeiss Axio optical microscope (Fig. 2). Spermatic cysts were observed, and mature cysts (see Pires, Silva & Bastos, 2014) were considered to indicate spawning proximity. Polyps that were empty during collection were considered non-reproductive.

### Fecundity measurement

Polyps with female and/or male gametes were considered reproductive, and only female polyps were considered to estimate fecundity. The total number of oocytes per polyp was determined following a methodology resembling that of Brooke & Young (2003), Goffredo, Arnone & Zaccanti (2002), and Smith & Hughes (1999). Colonies were considered as hermaphrodites if at least one polyp contained both gametes. Due to intraspecific variation in polyp sizes, fecundity (F) was evaluated by the relation between the total number of oocytes and the average size of the three reproductive polyps of the colony fragment. The fecundity coefficient (FC) equals the number of oocytes per reproductive polyp. This value was divided by polyp volume (in cubic millimeters). Fecundity values were obtained by the product of FC and polyp volume: F = FC/PV. The formula for cone volume calculated polyp volume (PV) as $\rm PV=\pi\times r^2\times h/3$ since polyps resemble cones.

### Data analysis

Data normality was assessed by the Shapiro-Wilk test (*p* < 0.05). As the null hypothesis was rejected, the analyzed random variables showed abnormal distribution. The Lilliefors normality test again evaluated this parameter, confirming the abnormal distribution of fecundity values. A log(x+1) transformation was applied to meet the variance analysis assumptions, and homoscedasticity was evaluated by the Bartlett’s test. After these *a priori* tests, parametric ANOVA statistical analysis and the *post-hoc* Tukey test were chosen for multiple comparisons of mean values. Correlations were performed *via* Pearson’s (r) coefficient. All analyses were carried out on the R language and environment for statistical computations on R Studio (R Core Team, 2015; RStudio Team, 2015) using the “ggplot2” package.

## Results

**Figure 3:**
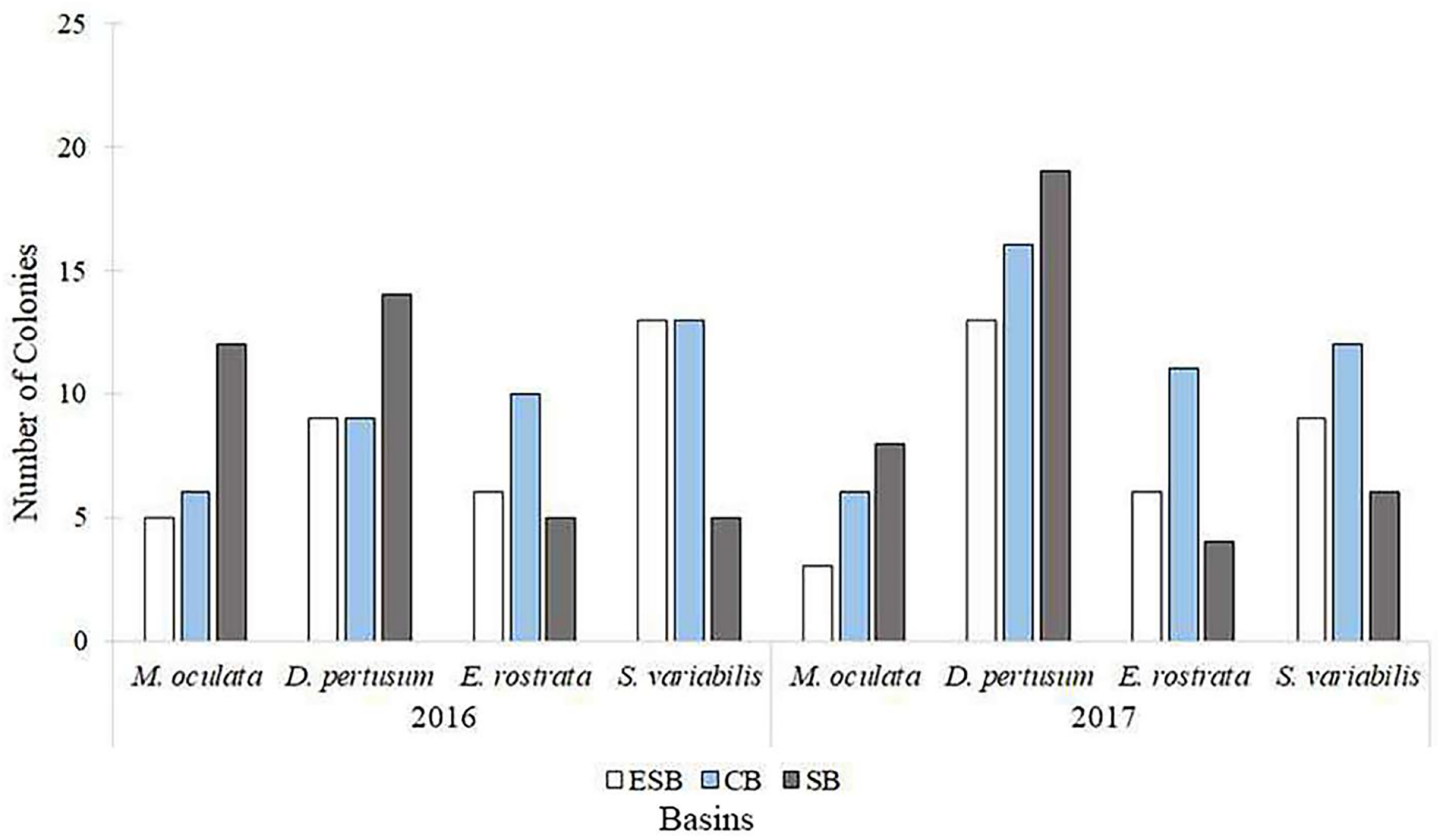
Number of colonies selected, among species, for histological analyses in each basin and year of collection. We analyzed specimens of all species in all basins in both sampling years. ESB, Espírito Santo Basin; CB, Campos Basin; SB, Santos Basin.

**Figure 4:**
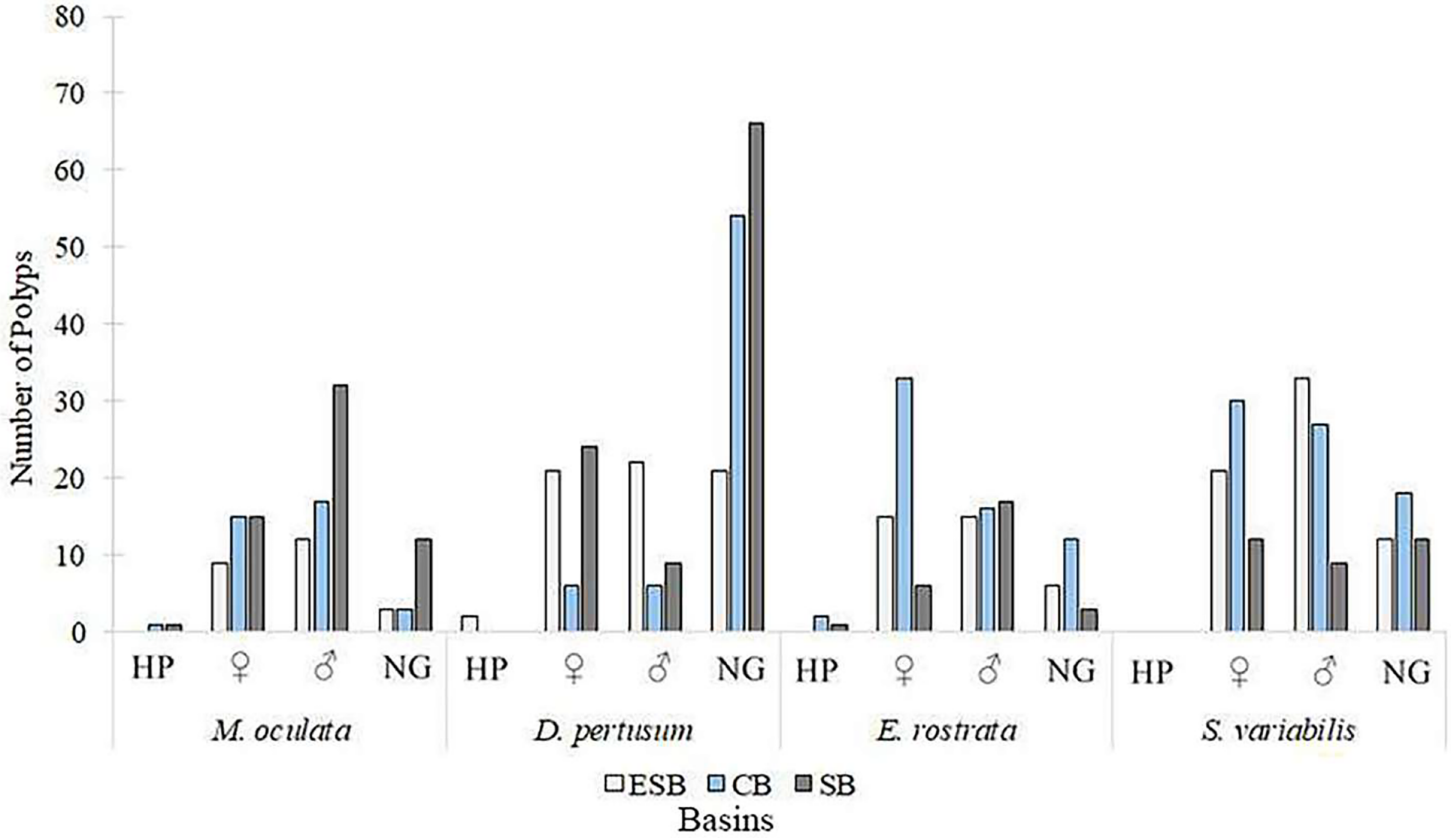
Number of polyps, among species, in different reproduction conditions. Polyps were classified into hermaphrodites, with oocytes, with spermatic cysts, and without gametes per basin. HP, Hermaphrodite polyps; ♀: Female; ♂: Male; NG: Without gametes. ESB, Espírito Santo Basin; CB, Campos Basin; SB, Santos Basin.

Of the 217 colonies (*n* = 651) of four species of corals collected in three basins and the two campaigns carried out by this study, we found that 142 were fertile. Of these, 68 contained spermatic cysts, *i.e*., 47.8% of samples were male. In total, 69 colonies contained oocytes (*n* = 207) 48.6% of all samples. We found that five colonies contained gametes of both sexes (3.52% of the sample). Only *Solenosmilia variabilis* sample showed no hermaphroditism. This study found other male polyps in fragments with hermaphrodite polyps. The number of colonies varied between species due to the need to search for colonies with female gametes. Depth ranges varied across species and basins due to the geographic distribution of coral mounds on the coastal margin of southeastern Brazil (Supplemental Material). Figure 3 shows the number of analyzed colonies per year and basin for each species. We found many *D. pertusum* samples without gametes, constituting the species with the most considerable discrepancy. Therefore, species required a larger sample size in seeking reproductive colonies. *D. pertusum* had few reproductive colonies in the CB. The main divergences between polyps by species referred to the proportions of male polyps and polyps without gametes. We also observed intra and interspecific behavior across basins. *M. oculata* and *S. variabilis* had higher ratios of male polyps in the SB and ESB, respectively, whereas *Enallopsammia rostrata* and *S. variabilis* had a higher ratio of female polyps in the CB and ESB, respectively (Fig. 4).

### Desmophyllum pertusum

**Figure 5:**
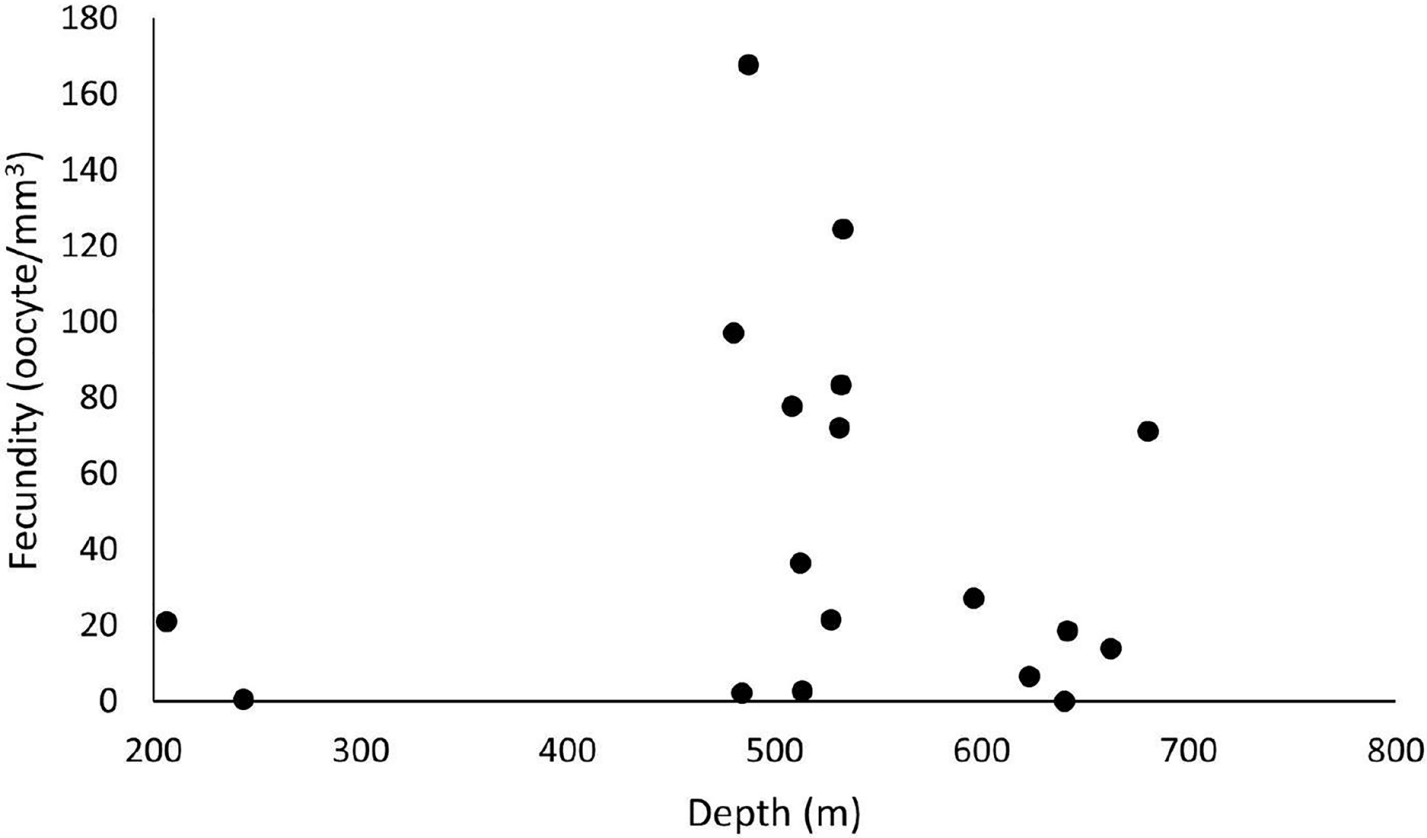
Fecundity dispersion by depth in 2016 and 2017 at ESB, CB and SB. Samples with female gametes occurred between 206 and 680 meters. The highest fecundity was noted at 487 m (F = 167.9 oocytes/mm^3^).

Out of 77 analyzed samples, 30 were reproductive colonies, meaning that 39% of the sample contained gametes, of which 17 were female (*n* = 51 polyps), 12 male (*n* = 36 polyps), and one hermaphrodite. Most polyps (88%) showed all three stages of oocyte development. Samples greatly differed in their number of oocytes. We found from 45 to 241,832 oocytes in the collected colony fragments, observing no significant differences across years and basins (ANOVA *p* > 0.05). However, considering the average fecundity between the basins, the ESB showed the highest average fecundity (F = 48.7 ± 19.9 oocytes/mm^3^). We found a single female sample in the ESB in 2016 (F = 2.95 oocytes/mm^3^), and four male colonies, whereas the average fecundity in the ESB exceeded that of others in 2017 (F = 55.2 ± 21.7 oocytes/mm^3^). We observed the second-highest average fecundity in the SB (F = 50.1 ± 16.7 oocytes/mm^3^). In 2016, it averaged 54.9 ± 21.8 oocytes/mm^3^ and in 2017, 46.9 ± 25.2 oocytes/mm^3^. In 2017, the mean total fecundity in the CB equaled 16.97 ± 10.3 oocytes/mm^3^, and we found no samples with female gametes in it in 2016. In the collections in late October, SB stood out with the highest fecundity; also, one colony was found in ESB and none in CB in the same period. We found the annual fecundity equaled 47.06 ± 11.4 oocytes/mm^3^. The mean per year totaled 47.51 ± 19.9 oocytes/mm^3^ in 2016 and 46.8 ± 11.8 oocytes/mm^3^ in 2017. We observed no significant differences in mean fecundity between years (*p* > 0.05). We found a maximum fecundity of 167.9 in the ESB in 2017 and a minimum of 0.1 in the ESB in 2017. We observed no significant differences in fecundity (ANOVA *p* > 0.05) between samples. However, a two-way analysis of the factorial interaction (year × basin) showed a significant difference (ANOVA F = 3.812, *p* = 0.0268). The Tukey test showed differences between ESB and CB in 2017 and between the significance thresholds for 2016 and 2017 (*p* < 0.05). We collected *D. pertusum* samples from 205 to 692 m depth, finding female gametes from 205 to 680 m (Fig. 5). The highest fecundity occurred at 527 m, followed by 533 and 480 m, from which point values show the lowest fecundity in our dataset, increasing slightly at 680 m.

**Figure 6:**
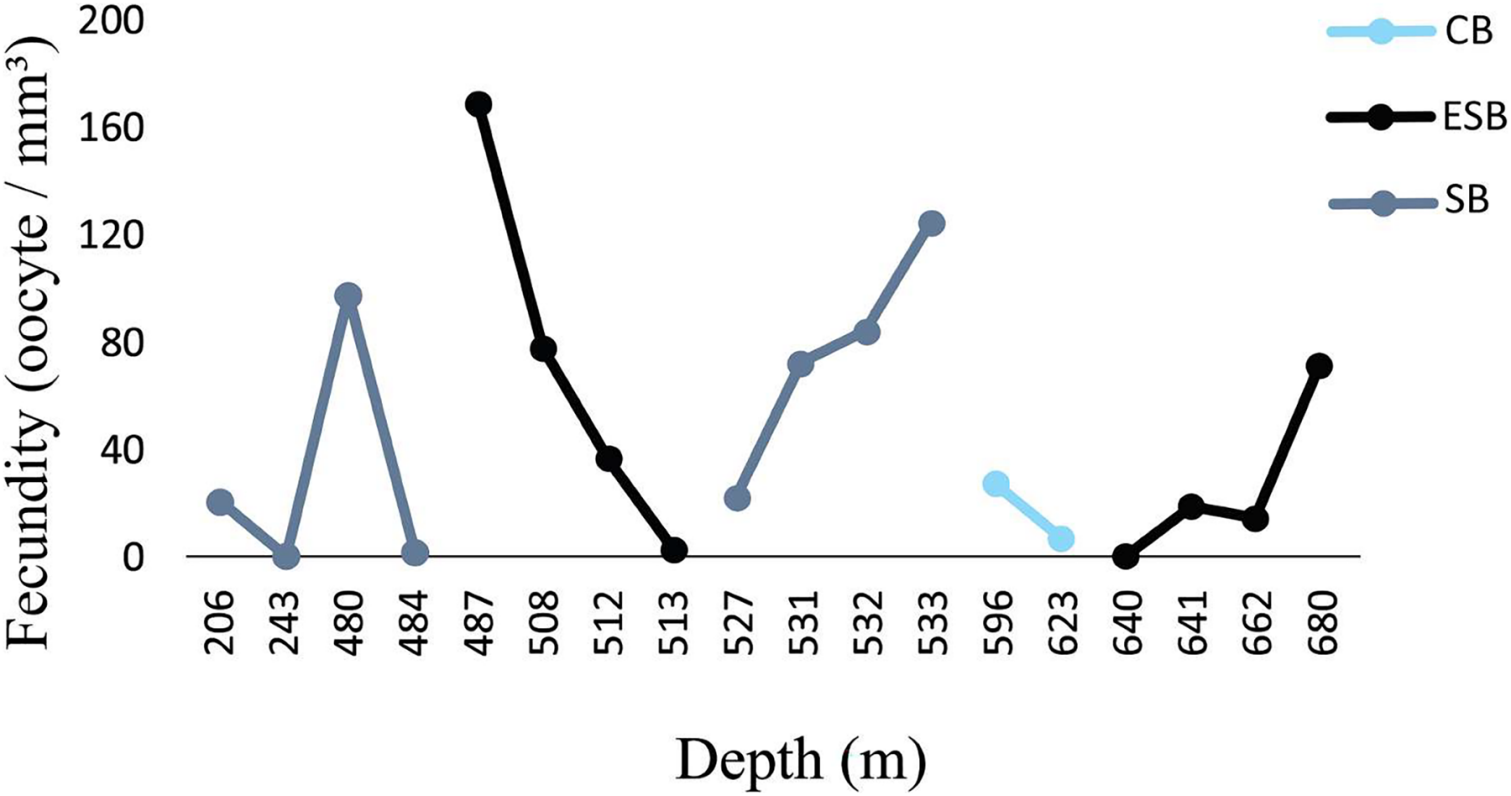
Fecundity along the depth gradient of different sedimentary basins. Few female samples were found in the CB. The ESB had fertile samples at greater depths (487–680 m). We observed a wide distribution of fertile samples in the SB, between 206 and 533 m.

**Figure 7:**
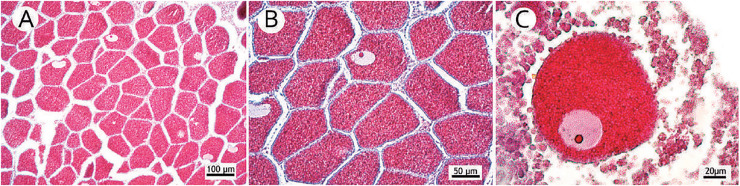
Occurrence of many small mature oocytes as a reproductive strategy. (A) Reproductive mesentery with a large number of oocytes stage III; (B) Detail of the close occurrence of oocytes stage III; (C) Highlight of a small oocyte stage III.

Figure 6 shows a higher prevalence of reproductive samples from 450 to 680 m depth. The SB was the only basin in which we collected *D. pertusum* colonies from 205 to 456 m and found lower fecundity at shallow depths (from 527 to 533 m). This basin also had the greatest range of fecundity by depth. Reproductive samples from the CB occurred only at 487 and 641 m. The depth of the samples from the ESB ranged from 456 to 692 m and its reproductive colonies, from 484 to 680 m, with higher fecundity from 487 to 513 m. *Desmophyllum pertusum* had the smallest mean oocyte size—97.5 ± 59.21 µm (Fig. 7). Its largest diameter equaled 230 µm. It also showed the largest average polyp size of all species: 229 ± 196.6 mm^3^.

### Solenosmilia variabilis

Out of 58 analyzed samples, 44 were reproductive colonies, meaning that 75.8% of the samples contained gametes, of which 21 were females (*n* = 63 polyps) and 23 males (*n* = 69 polyps). The entire species sample showed 74,189 oocytes, resulting in an average distribution of 3,532.8 ± 790 oocytes per colony fragment. The highest number of oocytes in a single colony fragment equaled 12,465, in the CB, in 2016. In 2017, another colony fragment in the CB contained 8,775 oocytes. Total average fecundity equaled 53.6 ± 10.7 oocytes/mm^3^, with the highest fecundity occurring at 200.7 oocytes/mm^3^ in the ESB in 2017 and the lowest, in the BC in 2016 (F = 2.45 oocytes/mm^3^). The highest average fecundity occurred in the ESB (F = 71.2 ± 26.2 oocytes/mm^3^), followed by SB (F = 53.3 ± 12.3 oocytes/mm^3^) and CB (F = 41.4 ± 12.3 oocytes/mm^3^). Comparing sampling years showed that 2016 had a lower mean fecundity (F = 44.6 ± 12.8 oocytes/mm^3^) than 2017 (F = 61.7 ± 16.9 oocytes/mm^3^). Collections took place at the end of the spawning period in 2016, resulting in more significant variability in fecundity between basins this year. Considering the mean fecundity between basins in 2016, we found only one female sample in the SB, which showed high fecundity (F = 78.3 oocytes/mm^3^), we also found three male colonies. The CB and ESB basins showed a mean total fecundity of 45.6 ± 19.1 and 31.2 ± 19.5 oocytes/mm^3^, respectively. In 2017, from highest to lowest average fecundity, the ESB, SB, and CB had 101.1 ± 38.9, 45 ± 12.8, and 34.8 ± 13.9 oocytes/mm^3^, respectively.

**Figure 8:**
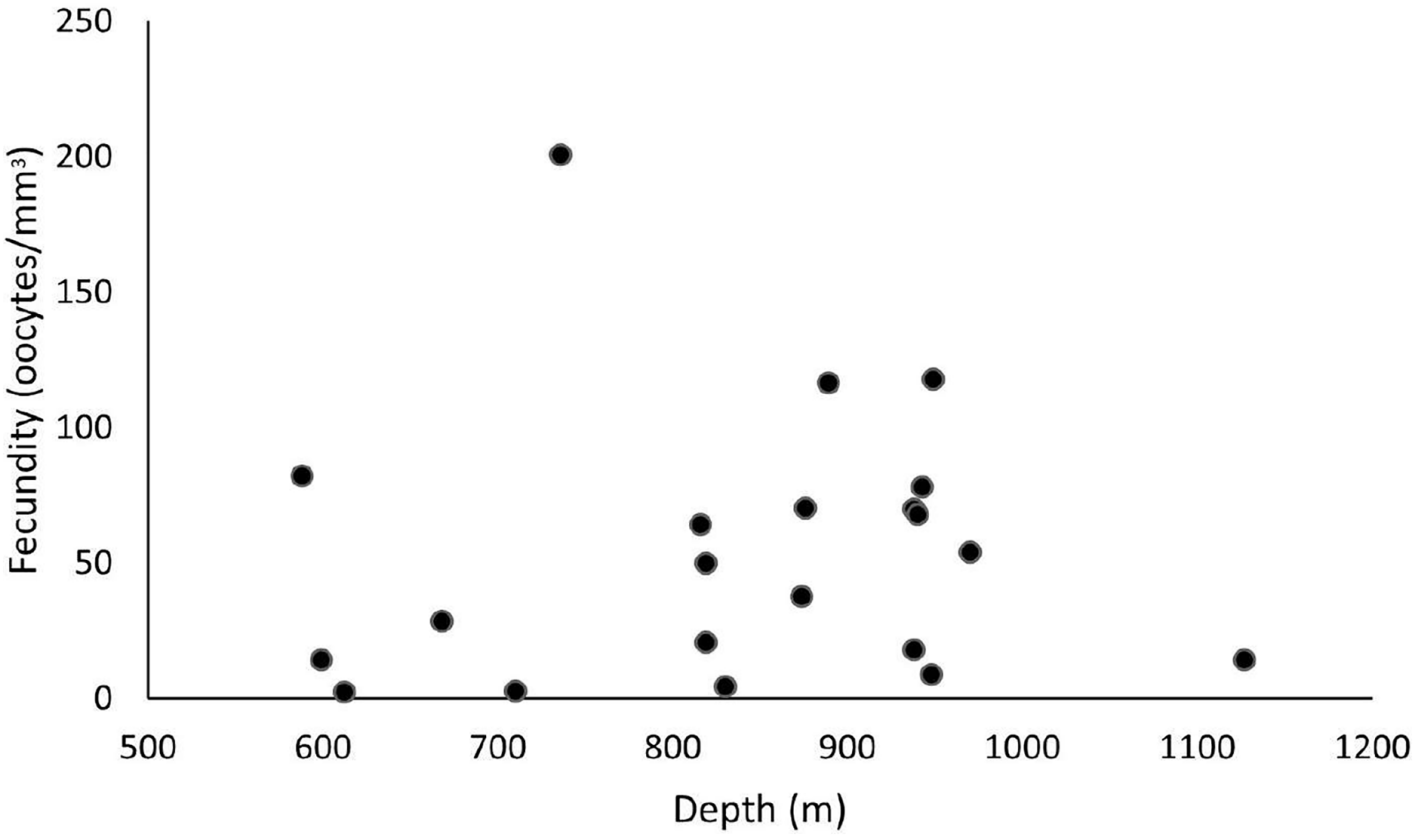
Fecundity samples along a depth gradient. Samples were collected between 588 and 1,127 m. The highest fecundity occurred in a sample at a depth of 736 m (F = 200.7 oocytes/mm^3^).

These samples occurred from 588 and 1,128 m depths, and the highest fecundity values occurred at 736 (F = 200.7 oocytes/mm^3^), 889 (F = 116.4 oocytes/mm^3^), and 949 m (F = 117.8 oocytes/mm^3^). The lowest values occurred at 612 (F = 2.4 oocytes/mm^3^), 710 (F = 2.7 oocytes/mm^3^), and 830 m (F = 4.6 oocytes/mm^3^) (Fig. 8). Nevertheless, we observed a higher occurrence of female samples from 800 to 1,000 m. Species showed a maximum oocyte diameter of 220 µm, an average oocyte size of 122.8 ± 9.4 µm, and an average volume of reproductive polyps equal to 21.5 ± 2.3 mm^3^.

### Madrepora oculata

Out of 40 analyzed samples, 34 were reproductive colonies, corresponding to 85% of the samples containing gametes, of which 13 were female, 19 were male, and two samples were hermaphrodites. We found 8,223 oocytes, averaging 548.2 ± 185 oocytes per colony fragment. The highest oocyte count in a single colony fragment totaled 2,663 in the ESB, in 2016. In 2017, the highest occurrence of oocytes equaled 1,384 in a single BC fragment. The average fecundity in all samples totaled 23.5 ± 7 oocytes/mm^3^, with a maximum fecundity (99 oocytes/mm^3^) occurring in the ESB, in 2016, and a minimum (0.06 oocytes/mm^3^) in the SB, in 2017. Species showed more significant reproductive effort in 2016, averaging 26.5 ± 12.2 oocytes/mm^3^ total fecundity. In 2017, the average fecundity equaled 20.2 ± 6.7 oocytes/mm^3^). The highest average fecundity among the basins occurred in ESB in 2016 (38.2 ± 30.5 oocytes/mm^3^), while the BS and CB totaled 28.9 ± 15.2 and 5.28 ± 3.74 oocytes/mm^3^, respectively. We did not find female samples in ESB basin in 2017. The SB basin had the second-highest average fecundity (F = 25.5 ± 9.2 oocytes/mm^3^) and CB, the lowest (F = 14.2 ± 5.9 oocytes/mm^3^). In 2017, SB showed 22.2 ± 13.5 oocytes/mm^3^, whereas CB obtained 18.7 ± 8.13 oocytes/mm^3^.

**Figure 9:**
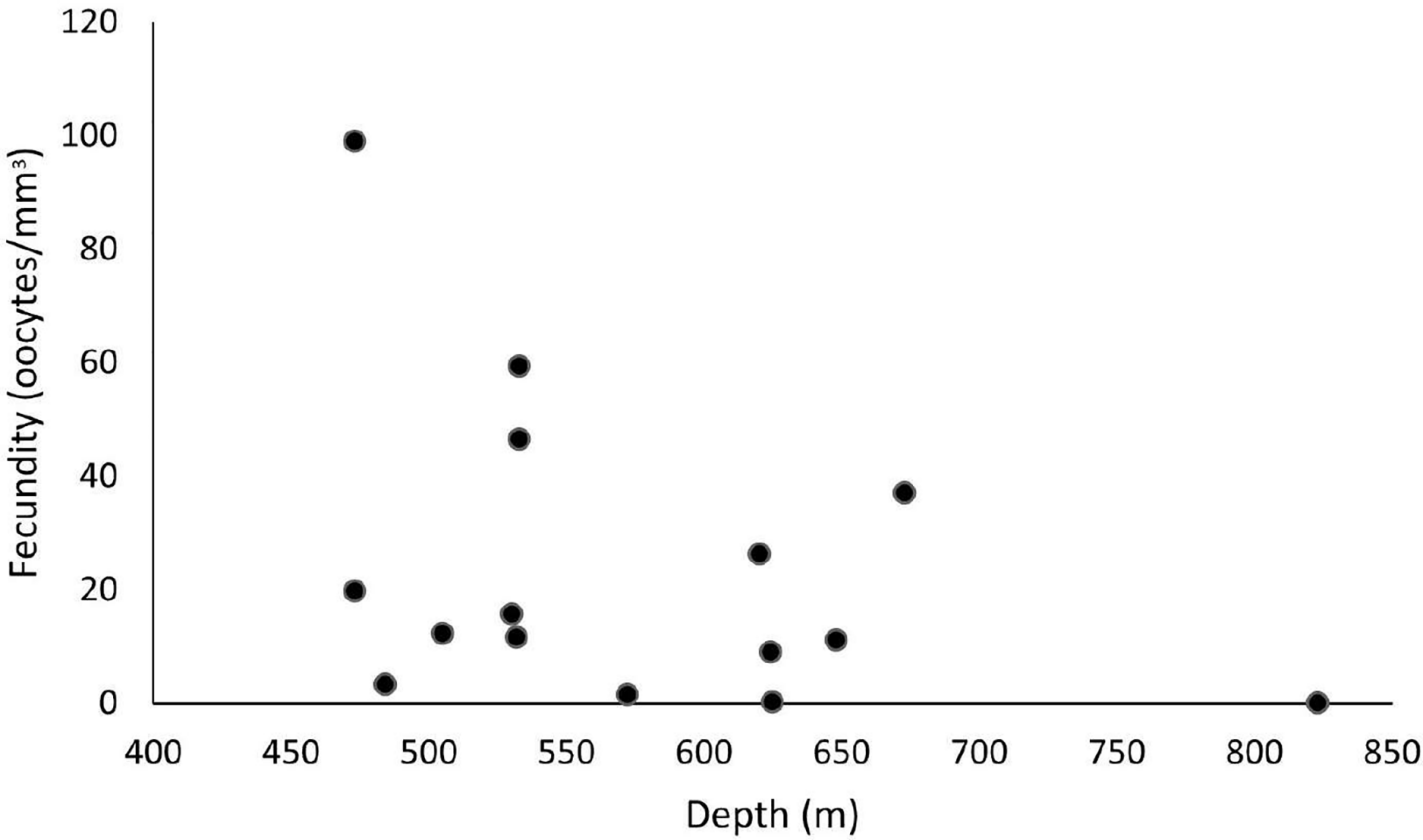
Fecundity along a depth gradient. Samples were collected from 465 to 892 m. The highest fecundity occurred in a sample at 473 m (F = 99 oocytes/mm^3^) depth.

Our samples ranged from 465 to 892 m in depth, and female colonies occurred from 473 to 823 m. The highest fecundity values occurred at 473 m (F = 99 oocytes/mm^3^), followed by 533 m (F = 59.4 and 46.6 oocytes/mm^3^) and 673 m (F = 37.1 oocytes/mm^3^). The lowest value occurred at 823 m (F = 0.06 oocytes/mm^3^), followed by 625 m (F = 0.24 oocytes/mm^3^) and 572 m (F = 1.54 oocytes/mm^3^) (Fig. 9). We observed maximum and minimum fecundity values in all basins. Furthermore, the species showed a 430-µm second largest mean oocyte diameter, a 183.9 ± 95.7 µm overall mean, and a 11.3 ± 7.25 mm^3^ mean polyp size (the smallest value).

### Enallopsammia rostrata

**Figure 10:**
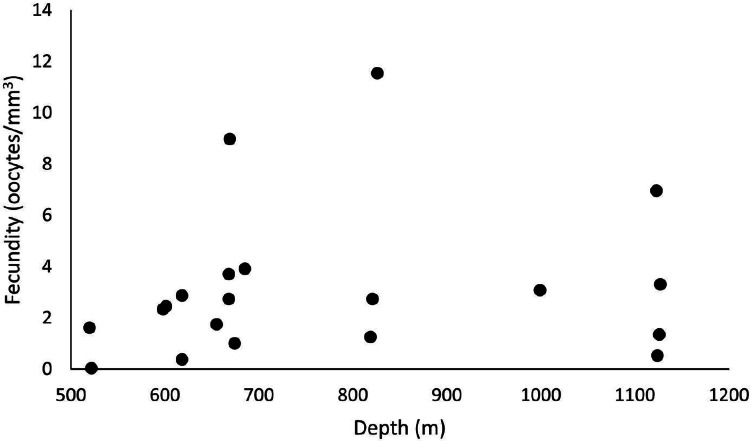
Fecundity samples along the depth gradient. Samples were collected between 486 and 1,128 m. The highest fecundity occurred in a sample at a depth of 826 m (F = 11.6 oocytes/mm^3^).

Out of 42 analyzed samples, 35 were reproductive colonies, representing 83% of the sample, of which 18 were female (*n* = 60 polyps) and 15 were male (*n* = 45 polyps). Only two samples were hermaphrodites. The samples totaled 5,457 oocytes, with an average distribution of 272.9 ± 47.7 per colony fragment. The highest oocyte count in a single colony fragment equaled 774 in the SB, in 2017, in a hermaphrodite sample. In 2016, the colony fragment with the highest number of oocytes totaled 464. The average fecundity in all samples equaled 3.1 ± 0.7 oocytes/mm^3^, with a maximum fecundity of 11.6 oocytes/mm^3^ in the SB in 2017 and a minimum of 0.03 in the ESB in 2017. In 2017, species showed greater reproductive efforts, with an average fecundity of 3.7 ± 1.18 oocytes/mm^3^ (unlike the 2.62 ± 0.7 oocytes/mm^3^ in 2016). The highest average fecundity occurred in the SB (F = 5.2 ± 3.21 oocytes/mm^3^), followed by the CB (F = 3.1 ± 0.74 oocytes/mm^3^) and the ESB (F = 1.97 ± 0.61 oocytes/mm^3^). In 2016, the CB showed the highest average fecundity (F = 3.12 ± 1.27 oocytes/mm^3^), whereas the ESB (F = 2.03 ± 0.35 oocytes/mm^3^) and SB (F = 2 ± 0.74 oocytes/mm^3^) had approximate values. In 2017, the SB showed the highest fecundity (F = 11.6 oocytes/mm^3^) stemming from a single sample, whereas the other basins showed the following averages: CB with F = 3.1 ± 0.9 oocytes/mm^3^ and ESB with F = 1.9 ± 1.8 oocytes/mm^3^. Our sampling ranged from 486 to 1,128 m in depth, and female samples occurred from 520 to 1,127 m. We found the highest fecundity values at 826 m (F = 11.6 oocytes/mm^3^), 669 m (F = 8.97 oocytes/mm^3^), and 1123 m (F = 6.96 oocytes/mm^3^). We observed the lowest values at 522 m (F = 0.03 oocytes/mm^3^), 618 m (F = 0.38 oocytes/mm^3^), and 1,124 m (F = 0.53 oocytes/mm^3^) (Fig. 10). The species showed the largest mean oocyte diameter of all studied species (377 ± 53 µm), with a maximum oocyte size reaching 900 µm and the second largest mean volume of reproductive polyps: 34.7 ± 6.8 mm^3^.

### Variations among species with continuous reproduction

We found no significant differences between species regarding sex ratio and non-reproductive polyps (ANOVA *p* > 0.05). Our bifactorial analysis detected no significant differences in total oocytes × year (ANOVA *p* > 0.05). However, only assessing species obtained a significant difference regarding total oocytes (ANOVA F = 12.13, *p* = 0.000046). the Tukey’s multiple mean comparison test found significant differences between *S. variabilis* × *E. rostrata* (*p* = 0.00011) and *S. variabilis* × *M. oculata* (*p* = 0.00093). We observed no significant fecundity differences between 2016 and 2017. However, our bifactorial analysis showed that species showing differing values (ANOVA F = 9.566, *p* = 0.0003). Our *post-hoc* evaluation corroborated how the fecundity of *S. variabilis* differed from that of other species (*p* = 0.0109) and the even greater difference between *S. variabilis* × *E. rostrata* (*p* = 0.0004). Fecundity according to basins evinced no significant differences (ANOVA *p* > 0.05).

**Figure 11:**
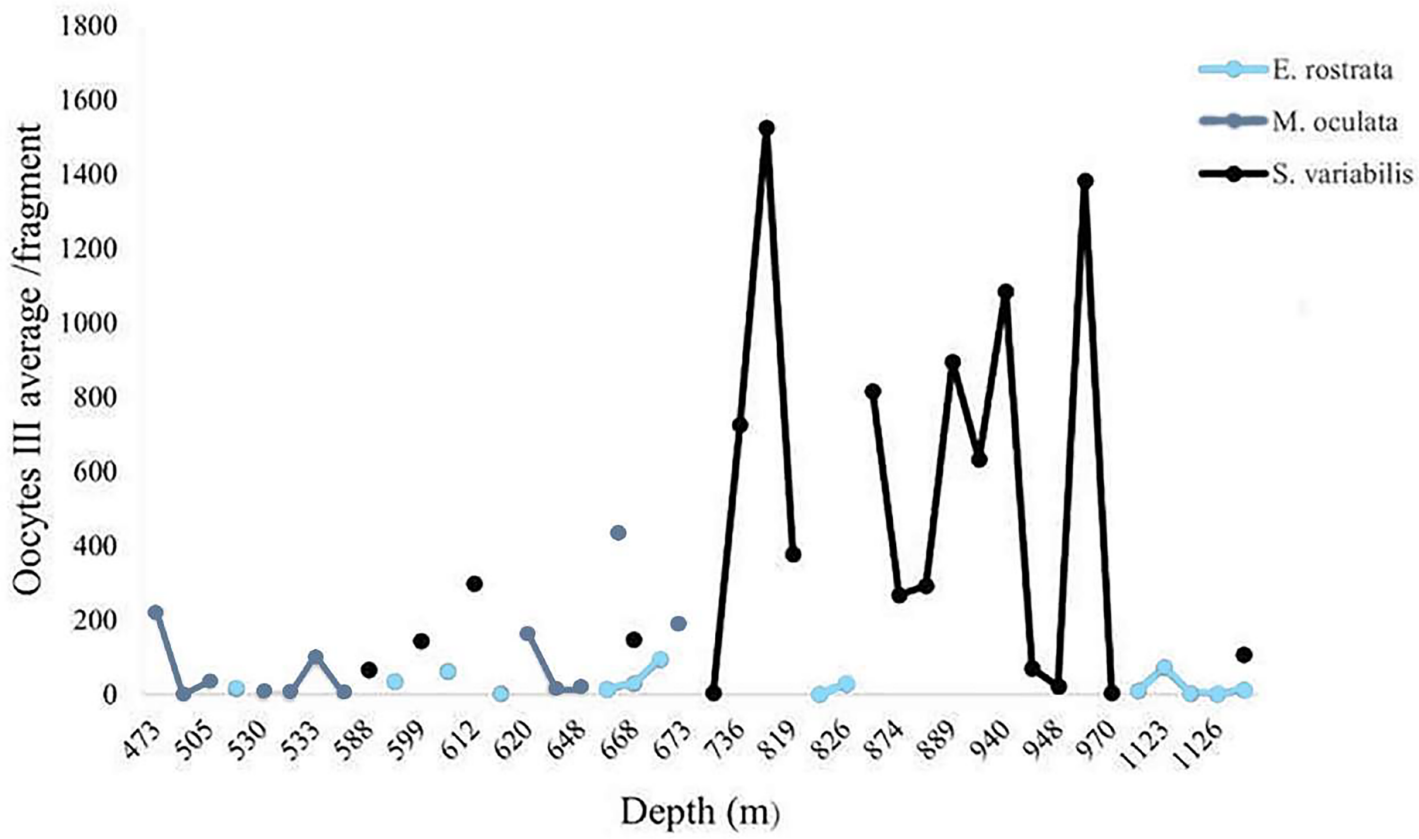
Oocyte III mean per fragment along a depth gradient. *Solenosmilia variabilis* showed the highest number of mature oocytes and the highest occurrence of reproductive female samples at greater depths, as did *E. rostrata*.

We calculated the average number of mature oocytes per colony fragment at different depths (Fig. 11) to evaluate the distribution of samples with female gametes at these depths. *Solenosmilia variabilis* showed greater mature oocyte averages from 736 and 949 m. Female *M. oculata* colonies occurred at shallower depths than the other species (up to 673 m) and *E. rostrata* spread evenly between depths, showing a low average number of oocytes.

## Discussion

This study observed that previtellogenic oocytes accumulate large amounts of yolk, forming lipid-rich vitellogenic oocytes. This observation aligns with the findings by Waller (2005) for deep-water species of corals, which resemble Fadlallah’s (1983) description of the oogenesis of shallow-water corals. We chose not to measure spermatic cysts, as they could provide inaccurate data on development due to their multicellular structures (Waller et al., 2023). According the authors, measuring oocytes has become the standard method to evaluate oogenesis and indirectly understand spawning periodicity. Such method only measures oocytes with a visible nucleus and nucleolus since histological sections require accurate diameter measurements. Furthermore, the presence of nucleolus ensures non-repetition.

The literature classifies the reproductive strategies of *E. rostrata* and *M. oculata* as quasi-continuous and continuous, respectively (Burgess & Babcock, 2005; Pires, Silva & Bastos, 2014). Corroborating this particularity, we found lower fecundity values when compared to other studied species. We can attribute this to several potential spawning events throughout the year that characterize species with continuous reproduction. Both species showed the majority of their reproductive colonies. Moreover, only 10 colonies of the entire sample of *M. oculata* (*n* = 5) and *E. rostrata (n* = 5) were non-reproductive. According to Pires, Silva & Bastos (2014), *S. variabilis* seems to show continuous reproduction, with a peak from April to September due to the high frequency of mature gametes, and particularly spermatic cysts. Our sampling period included the peak reproductive period reported by Pires and showed colonies with high fecundity rates. This species showed the highest overall average fecundity, compared to the others. Like *M. oculata*, it had more male colonies than females overall. In *D. pertusum* sampling, we found only one male colony in the CB from the 2016 collection (early September), and of the 16 colonies analyzed from the 2017 collection (late July), only three were reproductive and two presented oocytes. We did not find a good representation of fertile colonies in the basin, because the collection period differed from that reported by Pires (from May to July). Notably, the colonies analyzed at the end of July would have probably spawned, and those from September would not have yet started gametogenesis. On the other hand, the species demonstrated high fecundity rates at the end of May and in October, for ESB and SB, respectively. The average fecundity was equivalent in both years, and they were also similar between the Espirito Santo and Santos basins, with the latter being slightly higher. All samples contained oocytes in all stages of development. However, it should be noted that the SB colonies collected in October had more oocytes in stages I and II (*n* = 96,022) than oocytes in stages III (*n* = 26,622). These findings increase our understanding of the period of possible spawning on the Brazilian coast, and demonstrate the variations in different basins.

Despite the significant increase in research on deep-sea coral reproduction over the past 20 years, less than 7% of deep-sea coral taxa have data on reproduction. However, among deep-sea scleractinians, *D. pertusum* was extensively studied, including data on its sexual maturity (Waller et al., 2023). The oogenesis cycles show notable differences between populations in the Northeast Atlantic, including overlapping gametogenic cycles and continuous cycles with no gaps between the onset of gametogenesis and spawning (Brooke & Järnegren, 2013), as well a resting phase after the spawning period, with the onset of gametogenesis coinciding with the influx of food in the spring phytoplankton bloom (Waller & Tyler, 2005, Maier et al., 2020). Considering that biotic and environmental factors can influence the life history of a species, energetic relationships and reproductive strategies may vary between geographically separated populations, as in the case of the coral *Pocillopora damicornis* (Richmond, 1987). Waller & Tyler (2005) found that *D. pertusum* produced a relatively large number of small oocytes: 3,146 oocytes per polyp (±1,688) in August and 2,308 oocytes (±818) in October (with a maximum diameter of 140 μm). In comparison, *O. varicosa*, which also forms extensive reefs in deep waters of the North Atlantic, produced an average of 2,115 (±1,034) and 4,693 (±1,301) eggs per cm^2^ of skeletal surface area that also consisted of small oocytes (<100 μm) (Brooke & Young, 2003). The deep-water (1,300–1,799 m) octocoral *Anthoptilum murrayi* in the Southwest Atlantic produces comparatively large (up to 1,200 μm) mature oocytes in significant numbers—25,713–35,918 per colony, according to Pires, Castro & Silva (2009).

In the Northeast Atlantic, *D. pertusum* shows a year-long oogenesis, while spermatogenesis occurs in a shorter duration (Brooke & Järnegren, 2013). Colonies we collected from May to June produced significantly more mature oocytes (*n* = 328,988) than colonies collected in October (*n* = 11,177), corroborating the findings of Pires, Silva & Bastos (2014) on the spawning period of the species. Furthermore, we observed more stage I and II oocytes in polyps from colonies collected in early September 2016, indicating that the species initiates gametogenesis soon after spawning. Waller & Tyler (2005) also suggested that populations in the Northeast Atlantic (785–980 m depths) undergo a resting phase after spawning, initiating gametogenesis in late summer, which aligns with the influx of food and the spring plankton bloom. *D. pertusum* produces many gametes in a short spawning period, resulting in very small mature (vitellogenic) oocytes in fertile mesenteries. We can measure the large oocyte production of these species, but the different ways of analyzing fecundity can hinder comparative analyses (Waller et al., 2023). Our study indicates the high reproductive capacity of *D. pertusum*, given the high production of gametes. However, it is already known that global changes have affected environmental variability related to the development of organisms and the seasonality of physiological activities according to Walther et al. (2002). These changes increase metabolic costs and affect the reproductive capacity of these organisms (Maier et al., 2020). This may be the explanation for several colonies found without gametes in our sampling. On the other hand, the high number of *D. pertusum* oocytes in the reproductive colonies may suggest that the evaluated populations have managed to resist external factors that could influence their reproduction.

Another reproductive strategy involves a small number of large oocytes, as in *E. rostrata* and *M. oculata*. *M. oculata* had the lowest number of female samples of all species (37.5%), resembling that of *S. variabilis* (36.2%). However, the difference between both lies in the proportion of male individuals since *M. oculata* showed a higher proportion than *S. variabilis*, and fewer individuals without gametes. *Solenosmilia variabilis* showed greater reproductive effort in 2017 (with higher fecundity than in 2016, in which collections took place at the end of the spawning period). According to Waller & Tyler (2005), *M. oculata* produced a limited number of large oocytes, averaging 256 oocytes per cm^2^ of skeletal area, with a maximum size of 405 μm. We found that *E. rostrata* and *M. oculata* showed the highest average oocyte sizes. In a fecundity survey of 32 scleractinians, Harrison & Wallace (1990) found an inverse relationship between fecundity and egg size: species with eggs smaller than 250 μm in diameter produced from 1,000 to 10,000 eggs per cm^2^ of skeletal surface area. The large oocytes of *M. oculata* and *E. rostrata* we observed indicate the development of lecithotrophic larvae after external fertilization (Orejas & Jiménez, 2019). Pires, Silva & Bastos (2014) also observed the maximum diameter in *E. rostrata* (1,095 μm), *M. oculata* (650 μm), *D. pertusum* (242 μm), and *S. variabilis* (337 μm). Waller & Tyler (2005) proposed that *D. pertusum* produces lecithotrophic larvae based on observed oocyte sizes and reproduction timing. However, Larsson et al. (2014) argued for planktotrophic *D. pertusum* larvae as they can develop until they can settle without any observed feeding. This may also occur in *D. pertusum* in Brazil given the small size of its mature oocytes. The average size of *D. pertusum* oocytes in our study (97 ± 5.92 μm) was found to be smaller than the average described by Larsson et al. (2014) in a sample from the Northeast Atlantic (161 ± 4.06 μm). Waller (2005) described *D. pertusum* oocytes with a 140 μm average size and those of *M. oculata* with a 350 μm average size. As in Pires, Silva & Bastos (2014), we found few *M. oculata* and *D. pertusum* hermaphrodite samples. This study registered, for the first time, the occurrence of hermaphroditism in *E. rostrata*. Although gonochorism constitutes the most common reproductive pattern in deep-sea corals, previous research has found cases of hermaphroditism in several species (Waller et al., 2023).

Since fecundity estimates help to determine the recovery potential of a species (Waller et al., 2023), we suggest that this parameter highlights the recolonization potential of all species studied, given the incredible number of oocytes in the entire sample. Our results contribute valuable information about the life history strategies of key deep-sea habitat-forming corals. This study stemmed from the findings of Pires, Silva & Bastos (2014) regarding the gametogenesis and temporal patterns of reproduction that lead us to evaluate fecundity during peak reproductive periods. The collection region has habitats with great spatial complexity and biodiversity that support several marine communities of significant economic interest. Our sampling provided an overview of the reproductive effort of deep-sea coral species, enabling comparisons of fecundity data for each species across several periods, depths, and locations. Since, anthropogenic changes and pollution could impact this deep-sea ecosystem, it is necessary to highlight the need for further research—especially physiological assessments of corals—to evaluate the state of the major deep-sea framework-forming coral mounds (Orejas & Jiménez, 2019). Thus, future research should aim to better understand the environmental factors influencing gametogenesis processes and larval development to further our understanding of deep-sea coral reproduction cycles and ecological strategies.

## Conclusions

This study showed that more than 63% of all samples contained gametes, indicating a good reproductive sample occurrence in all species. We observed neither significant annual variation in fecundity rates between species nor significant geographic variation between the reproductive efforts of each species across the chosen sedimentary basins. We report for the first time the occurrence of Brazilian *E. rostrata* hermaphrodite colonies. This study explored a wide area in the Southeast Atlantic, expanding the knowledge of reproductive biology of its main deep habitat builders. The influence of external factors on reproductive processes remains a gap in the scientific knowledge. Therefore, future research should focus on better understanding the influence of these factors on reproductive processes.

## Supplemental Information

10.7717/peerj.19525/supp-1 Supplemental Information 1 Raw sample data. All histologically processed samples, oocytes found per colony, calculated fecundity, and polyp volume were calculated. Samples were analyzed separately and together.

## Additional Information and Declarations

### Field Study Permissions

The following information was supplied relating to field study approvals (i.e., approving body and any reference numbers):

Authorization for the collection and transportation of biological material was granted by IBAMA (No. 731/2016).

### Data Availability

The following information was supplied regarding data availability:

The raw measurements are available in the Supplemental File.
